# Clinical Lecture

**Published:** 1880-12

**Authors:** Austin Flint

**Affiliations:** Proffessor of Principles and Practice of Medicine in Bellevue Hospital Medical College


### CLINICAL LECTURE. PLEURISY, PNEUMONIA; DISEASE OF THE HEART.

By AUSTIN FLINT, M.D.,
Proffessor of Principles and Practice of Medicine in Bellevue Hospital Medical College.

*Gentlemen:*—I have selected several cases to-day for our clinical study, and I can say what I have said in my previous lectures this Spring, that the cases are new to me. With one exception I have not seen the cases that I will bring before you to-day. It shall not be the case hereafter, because I go on duty to-morrow, though, perhaps, with reference to our clinical study it is not a disadvantage.

I shall first ask your attention to this patient. He gives the following history: His name is William G., forty-two years of age, a laborer, and a native of England. He was admitted into the hospital yesterday. The family history is unimportant. He had yellow fever sometime ago; with this exception he has enjoyed good health. Two weeks ago he slept on the dock all night. The next morning he felt chilly. He had a pain under the free borders of the ribs on the left side. Soon after he began to cough, with a scanty expectoration, and soreness over the chest on the left side. He also passed some blood with his stools. On his admission he still complained of pain in the left side with soreness of the chest, and with cough.

Well, let us stop here, gentlemen, and see, with these facts before us, what we are to look for. We should not form an opinion from an insufficient number of data. That is an important practical precept to bear in mind with reference to diagnosis, and I think it is not rare for the rule to be violated. We see a patient, we get a certain number of facts from his history, we form an opinion at once prematurely. Well, now, the disadvantage of that is this: As soon as we have formed a conclusion we are on the ground of a party who is endeavoring to sustain that conclusion by further facts which may be brought out and that not infrequently leads to error of diagnosis. It is, therefore, an important precept that we should wait until we get all the facts before we bring our minds to any conclusion, as to diagnosis. Still, of course, when we get the facts out to a certain extent we form an idea what we are to seek for; not to form an opinion as to what exists, but what we are to look for.

Well, now, here we have an exposure, a lying out of doors, perhaps under the influence of alcohol. It is to be suspected certainly. It is not likely a man would lie out of doors all night for the mere pleasure of the thing, and it is not a necessity, because a person can get in doors some-where, so that I think it is fair that he had been taking a little something—he tells me he had. After that exposure, the next morning, he finds he has a pain in the left side, and soreness, and some cough. Well, this pain following that exposure and the soreness bring before our mind especially two things, pneumonia, pleurisy. How about the two, looking at the symptoms that are brought out. A patient with pneumonia is obliged generally to take the bed, but not always. We occasionally meet with pneumonia in the second stage in dispensary practice. A patient came to see me last week who had ridden two or three miles the preceding day, thinking the ride perhaps would enable her (she was a young woman) to shake off the disease, and I found, when she came to see me, that she had a temperature of 103°, and the physical signs of pneumonia in the second stage, but these are exceptional instances. The patients are obliged generally to take to the bed with pneumonia, whereas, with pleurisy they are not, necessarily, it depending upon the acuteness of the disease. Sub-acute pleurisy does not compel patients to take the bed, so that so far as we have gone in the history we would look for pleurisy rather than pneumonia And now what are the physical signs as recorded here?

"Yesterday there was dullness on the left side, with diminished respiratory sound; with loss of vocal fremitus and vocal resonance."

Well, that tells the story. Over most of the left side there was dullness in percussion. That would not enable us to discriminate between pleurisy and pneumonia, but with that there was diminished respiratory sound and a loss of vocal resonance, a loss of vocal fremitus Now liquid effusion incident to pleurisy, which occurs in the majority of cases of primary pleurisy, produced these signs, whereas, pneumonia, if it had led to consolidation enough to give rise to dullness on precussion, would have given us either bronchial respiration or broncho vesicular respiration and bronchophony, or increased vocal resonance. So you see how those signs mark the differential diagnosis.

But we need not be content even with these physical signs, although they are conclusive. I find it stated here that clear serum was drawn off with the syringe. That is a hypodermic syringe was used, and a little clear serum was drawn from the pleural cavity which substantiated the diagnosis of pleurisy with effusion, and moreover showed that it was a case of simple pleurisy; that it was a pleurisy with serous effusion and not empyema. Now, we will not stop a great while with this case. It is a simple case, yet without a knowledge of certain things it would be a case that would involve difficulty in diagnosis.

We will now make a physical examination. The left side is the side on which we get pleurisy oftener than on the right. Over the upper left lobe we get vesicu'o-tympanitic resonance. It just entered my mind that this would be a good case on which to make an observation with regard to the point which I spoke of last winter. In speaking of the physical diagnosis of effusion I have been accustomed to say that one of the points is, that the line between flatness and resonance is a horizontal line on either the anterior or the posterior aspect of the chest. I have not been accustomed to say that it was a horizontal line all around the chest of the affected side; that I know it is not But that point was taken up and criticised. Now let me see how we find it here. I think the criticism was not correct. It is not often a horizontal line on the posterior aspect of the chest. It certainly is not in all cases. Now let us see how we get it here.

In this case, anteriorly, I think we are able to say that a horizontal line does indicate the level of the liquid. Now let us see if we can examine behind. It is not quite so easy to make the examination behind, because we have more bone and muscle. We find that it is not a horizontal line. It is a curved line.

Well, now, what we have to verify here is, that below this line, and above it too, probably, we have diminished respiratory sound, probably absence of respiratory sound here below, unless the respiratory sound above is transmitted downward, or, what more frequently occurs, the respiratory sound on the other side is transmitted laterly. I perceive no respiratory murmur here. He has not a great deal of fremitus on either side, but the absence of respiratory murmur, the great diminution, almost absence of vocal resonance here on the one hand, and the fact that we do not get any bronchial respiration and bronchophony, shows that what we find here on percussion is due to the presence of liquid.

What are the indications for treatment in such a case as this, and what the prognosis? The prognosis favorable; he will get well. There is no reason to doubt it at all.

How shall we treat him? Shall we put him upon hydragogues and diuretics with a view to get rid of the liquid effusion? Well, it is not necssary to do much in these directions The liquid will absorb, perhaps rapidly. Certainly very active hydragogues or diuretics are not called for. I should rather be disposed to have him keep warm, comfortable, give him good nourishment, and not do much of anything, because it will tend to get well of its own accord. Shall we take out the liquid? There is no need of it. There would be no great objection to doing it, but there is no need of it. You see there is not enough to embarrass him in breathing. In short, the case is one which, if there existed no pulmonary disease before, as we have reason to think there was not, will tend intrinsically, and probably in a short time, to get well But we could help matters perhaps by giving him some diuretic remedies. I should resort to those rather than to hydragogues, and the blister which I see has already been prescribed for him is a good thing. I do not exactly understand the *modus operandi* of blisters over the chest in promoting the absorption of effused liquid, but observations seem to show that they do have that effect, and it is pretty much the custom when blisters are not employed to paint the chest with iodine. My own belief is that we do not accomplish much therapeutically by that application ; but it is a very excellent application with a view to moral effect. When patients see that they are colored yellow by this application they feel as if something is being done that is pretty potential, and it makes the mind easy, comfortable. So much then for that case.

Case II.—The next case that I shall present to you is a case of pneumonia, so that we shall have the opportunity of studying now in succession first, a case of pleurisy, and then a case of pnemonia.

His name is Henry D., twenty-one years of age, a laborer, a native of the United States, and was admitted on the twenty-seventh of April. The family and previous history of the patient having no bearing on his present disease. On April 23d, the patient had a severe chill. Now mark the contrast. In the first case there was a chilly sensation, but no pronounced chill. Pneumonia, as a rule, commences with a pronounced chill. Acute pleurisy is accompanied at the outset with some chilly sensations, but exceptionally with a pronounced chill. This was followed by cough and an expectoration of bloody sputa. In pleurisy, a cough with little or no expectoration, and what expectoration there is simply mucus, produced by cough. He had intense febrile pneumonia, and considerable dyspnœa, together with complete anorexy, and rapidly supervening exhaustion. Here now we have a disease of a different character ; fever, prostration, obliged to take to the bed. We have in fact there not a local inflammation as in the last case partly, but a fever with a local affection characteristic of it.

On the 25th of April, he began to have severe pain, the pain occurring rather late you see, two days after the symptoms denoting fever. Let me call your attention to this fact, because it is of some interest. Here the febrile phenomena made their appearance before marked symptoms denoting that there was inflammation in the chest. This pain was short, lancinating in character, and situated about the left nipple, the pain in character being the same as in pleurisy, but instead of being diffused it is comparatively limited, its seat being about the left nipple, and aggravated by the act of respiration and cough. All of these symptoms persisted up to the time of the patient's admission, which was on the twenty-seventh, four days after the date of the attack. On the twenty-eighth the patient's face was flushed, the tongue was coated, the sputæ were rusty, and viscid—the characteristic sputa of pneumonia. The cough and the pain in the side persisted, and the pulse in the morning was 110, the respirations were 38, and the temperature 103°. In the afternoon the pulse was 115, the respirations 36, and the temperature 102 75°—well the pulse was more frequent than in the morning, the respirations were a little fewer, and the temperature was a little less, not much difference however.

Now physical examination showed dullness over the left lower lobe, with bronchial respiration and bronchophony. The respiration over the left upper lobe and the entire lung was exaggerated. Other thoracic and abdominal organs normal. The urine was high colored, its specific gravity 1020, and otherwise normal.

On the 19th, yesterday, in the morning the pulse was 80, 110 the previous morning; the respirations 35, 38 the previous morning; the temperature 99°, 103° the previous morning. In the evening the pulse was 76, the respirations were 52, and the temperature 98°. All these symptoms indicate convalescence and resolution, accompanied by broncho-vesicular respiration, increased vocal resonance, and rales redux have commenced.

Now, gentlemen, here we have very concisely, the whole history of the case. The patient was attacked on the 23d, entered the hospital on the 27th, four days afterwards. After his attack he went to the dispensary and got some medicines. He does not know what they were, but some cough mixture, something palliative. I suppose that at that time the symptoms and signs hardly showed the character of the disease. We may say virtually he had no treatment. The patient has not been under my care, but I am told that he has had no potential treatment. He has had the spirit of mindererus, a palliative remedy. He has had a little quinine, but not in any full doses. I wish he had not had it. I doubt whether it had any influence in this case, but we can not say positively that it has not. Quinia does have an influence on pneumonic fever, and in certain proportion of cases causes it to resolve, but I do not think this patient has taken enough of it to have much effect. But I can not say positively what I would have said if he had not taken quinine. That here we have a good example of the natural history of this disease. I do not feel at all unwilling to think that the quinia may have had an influence I have seen it administered in this disease and I feel perfectly sure, as I have just said, that it arrested it or rendered it abortive, but I will repeat that I do not think this patient took enough of it, so that we must leave that a somewhat unsettled point whether this sudden change in his condition was the result of his treatment in any measure or whether it was simply the natural course of the disease. The disease does pursue this natural course in a certain proportion of cases.

He entered on the 27th; on the 28th he had a good deal of fever; the pulse 110, the respiration 38, the temperature 103°, whereas on the 29th in the evening his pulse was 76, the respiration 32, and the temperature 98°; he is now convalescent then, that is on the 29th; he was attacked on the 22d, resolution taking place on the sixth day.

Now let us see what signs he has, and then we will not trouble him any further. We ought to have on the left side vesiculo tympanic resonance over the upper lobe. I should think we had. This is a good example of it. The lower lobe being more or less consolidated we get an increase of intensity, and elevation of pitch, and a tympanitic quality, which are the qualities of vesiculo tympanitic resonance. Now we ought to have in whatever position this patient may be, sitting or recumbent, an oblique line here, above which we get this vesiculo-tympanitic resonance and below which we get either flatness or dullness, unless the stomach should happen to contain gas in considerable quantity, and then we might get the vesiculo-tympanitic over a purely tympanitic resonance. We can mark out the oblique line here without difficulty. When I come to auscultate posteriorly I find the lung almost entirely resolved. Now here within three days, the physical evidences have shown when the patient was admitted that there was complete consolidation, bronchial respiration, bronchophony, those signs have nearly all disappeared, so that here we have an instance of very rapid resolution which sometimes takes place in cases of pneumonia. There is a very considerable difference in different cases with respect to the length of time connected with the stage of restoration. Sometimes it is as rapid as it is here, and in other cases it is very slow, occupying sometimes even weeks before it is completed, and that we have learned since the time of Læennec. When Læennec began to study by means of auscultation cases of pneumonia, the natural duration of the disease was unknown. It was supposed that pneumonia was a disease that would generally destroy life unless treated very energetically, and Læennec was in the habit of giving antimony, adopting a treatment which originated with two Italian physicians of giving antimony in large doses, producing after awhile a degree of tolerance by the stomach of large doses in order to do good. And in his work on auscultation he is very enthusiastic as to the effect of antimony in promoting resolution in the second stage of pneumonia. Now, had he the benefit which we have acquired since his day, of a knowledge of the natural history of the disease, he would have to modify his opinion very essentially, because suppose in this case we had not known the natural history of the disease, and I should say I had a remedy which had a very special influence in promoting absorption of exudation in pneumonia, I could have brought in a patient of this kind and triumphantly pointed to it as an evidence of the marked effect of that remedy.

Case III. Now I have two cases of disease of the heart I propose to present to you and make the subject of study if time permit, valvular disease and enlargment of the heart. In the case which I will first present, I have not seen the patient. I read the history for the first time now.

This patient's name is Charles K , aged 36, a cook by occupation, a native of Germany. He was admitted on the 9th of January, last, so that he has been in the hospital for some time, as you see. The family history is unimportant. The patient has led a comparatively regular and temperate life. He has been obliged to work hard, in a hot kitchen. He has been accustomed to the moderate use of wine and beer, but has avoided spirituous beverages. He had gonorrhœa years ago, but positively denies ever having had syphilis. He says he has suffered at times from sub-acute rheumatism, but he gives no history of the acute, articular variety. That is a point of interest. While there is greater liability to cardiac complications in acute articular rheumatism, that liability is by no means wanting in sub-acute rheumatism, a very important practical fact to bear in mind, because an articular rheumatism, or rheumatic fever is sub-acute, we must not feel easy and let the patient go without protective treatment against cardiac complications.

Three years ago the patient's legs became greatly œdematous. He suffered also from slight dyspnœa, from pain and tenderness in the right hypochondrium, and from impaired digestion. These symptoms did not incapacitate the patient for labor, and they soon disappeared. Mark that. Patients with rheumatic affections of the heart not infrequently suffer for a brief period from the effects of it in the way of dropsy and dyspnœa. But their condition improves while, of course, the rheumatic affection does not change. But their condition in other respects improves and these symptoms disappear. About two years ago the same symptoms again developed themselves with the addition of cardiac palpitation, and a troublesome, persistent cough associated with marked dyspnœa. These symptoms continued only a few weeks—another instance of what I have just said. Six and a half months ago the patient again noticed œdema of his face, and this was followed by œdema of the feet and legs, and by marked dyspnœa on the slightest exertion. The patient had a cough but expectorated only mucus. He had severe frontal headache, pain over the region of the liver, constipation, disordered digestion, nausea and vomiting. His appetite was fair and the tongue normal.

Now, I do not know what we will come to here, but I suspect we have evidence of something more than cardiac trouble. On admission, January 9th, the patient presents the symptoms enumerated above. He was anæmic, his prolabia were at times cyanotic, the breathing labored, the cough paroxysmal, the sputa scanty and mucus in character. The urine was of light color, specific gravity 1016, and normal in its chemical reactions and microscopical appearance. Well, I read that with some surprise, for I thought we should find from the previous history evidences of renal trouble, but it seems not, at that time at any rate.

Now on physical examination—mark these points which we will soon verify—the precordial space was enlarged and the apex beat removed some distance to the left of the mammary line. The impulse was visible over an enlarged area, but not increased in force. Now here is evidence of enlargement of the heart and something more, that the enlargement of the heart involved either weakness from some change in the muscular walls, or I may say more probably enlargement with predominant dilatation.

A loud musical murmur was heard with the first sound of the heart—it was then either mitral regurgitant, an aortic direct, or a tricuspid regurgitant—it lies between those three. Its greatest intensity was at the apex, and it was continued to the left of the apex, and heard distinctly near the lower angle of the scapula. It was then a mitral regurgitant murmur. Another murmur was heard with the first sound at the base in the second intercostal space of the right side, and was continued into the carotids. There we get the evidence of an aortic direct murmur. This patient, therefore, had a mitral regurgitant murmur and an aortic direct murmur, both being systolic.

During the patient's sojourn in the hospital he has suffered from paroxysmal attacks of dyspnœa, attended by cough. He has also persistent cephalalgia and œdema of the face and lower limbs.

Well, gentlemen, you see that he is pallid; the prolabia have a slight appearance of cyanosis; the lower limbs are œdematous, but not greatly so. They have been much more so. Now let me verify these signs without going into a very full explanation of them. That I presume for many of you is not necessary. There is the apex, which is as low as the seventh intercostal space, and is at least an inch and a half removed to the left of the nipple line. Its normal situation should be in the fifth intercostal space, and a little within that line. We have then that evidence of enlargement. The impulse is not strong; I perceive it distinctly, but not strong. Now, then, we should expect to find an area of precordial dullness corresponding to that. Yes, the area is increased, and the degree of dullness is increased. We have then the evidences of enlargement very readily seen.

Now, then, if I put a stethoscope here I ought to hear a murmur with the first sound of the heart, which I do. It is very distinct, bellows in character now, and is transmitted from the apex to the left as far as I examined. It is also heard, as the report says, behind on the left side at the lower angle of the scapula. That is a soft blowing murmur, tolerably loud. Now, at the second intercostal space, at the right of the sternum, I get another murmur, it is not as loud as the first, but it is not the same murmur, though heard with the same heart beat. It differs in character. It is high in pitch comparatively; the first low in pitch. I know, therefore, that we have a mitral regurgitant and an aortic direct. Between the points mentioned I get both murmurs very distinctly heard. Those are the only murmurs that I can discover. He has not a presystolic; he has not a tricuspid direct; he has not an aortic regurgitant.

Now, let me direct your attention to one thing: I want to determine whether this mitral regurgitant murmur denotes much regurgitation. This murmur does not give me any information with regard to that. A small regurgitant stream may give a much louder sound than a larger stream, but if there be much regurgitation two things will follow. In the first place the left ventricle will send into the aorta a quantity of blood diminished by just as much as regurgitates. And then again, in proportion as there is an abundant regurgitation the effect will be marked upon the right side of the heart, and we should be likely to have hypertrophy of the right side of the heart. Now both of those things will affect the second sound of the heart as it is produced in the aorta and pulmonary artery. I will listen here to get the aortic second sound. Now if a good deal of blood regurgitates through the mitral valve, and a corresponding less amount is sent out of the heart, this sound will be weak; the aortic second sound will be proportionately weak. The right ventricle being hypertrophied, that will intensify the pulmonary sound which I can hear. The second sound of the heart is produced by the aortic and pulmonary valves. By putting the stethoscope here I get the sound produced by the aortic valves; and here, by the pulmonary, if I find the aortic second sound feeble and the pulmonary comparatively loud, I shall get evidence that the mitral regurgitant is considerable in amount. It is very marked indeed. The aortic second sound can scarcely be perceived, it is very feeble; the pulmonary second sound, on the other hand, is quite loud, louder than normal. So that I feel warranted in saying that there is regurgitation, and the right ventricle is hypertrophied.

So much then for the history of this case as regards the diagnosis. Now, I think I will hardly have time to get through with the other case, and I will make some further remarks on this patient. I think I will reserve the other case, which, by the way, is an excellent case to bring in contrast with this, but I would not have time to do justice to the physical signs and the history, so I will leave it and make a few remarks about the causation, the indications for treatment and the prognosis in this case.

Undoubtedly in this case the cardiac affection is to be dated from the rheumatic endocarditis. Valvular lesions of the heart in a great majority of cases originate in that way, and we have seen in the history that this patient has had sub-acute rheumatism at different times. When he had the attack of rheumatism, which was complicated endocarditis, I am, of course, unable to say. I might perhaps by diligent inquiry, but that is not so very important. Now, that endocarditis left behind it certain effects, there was an exudation on the valves which was not washed away, but remained there. This slowly led to changes by which the mitral valve became insufficient, either contracted or possibly attenuated, and rupture may have taken place. At all events some one of the various forms of lesion which give rise to insufficiency of this valve occurred. We do not get the physical sign, the murmur which indicates contraction at this orifice. Now, one point is, that this change in the valve has been going on for a long time; for many years. How many I cannot say, but a number of years. For a considerable length of time the patient experienced no great inconvenience from it. You see the history says that three years ago he first noticed œdema and slight dyspnœa. Now, this affection had existed for a long time before that. It had existed long enough for this regurgitation to produce changes in the heart, to produce an enlarged heart, and not only an enlarged heart, but a heart enlarged by dilatation as well as by hypertrophy. Well, he had the effects at that time, the œdema, the dyspnœa, of disease of the heart characterized by mitral lesions and enlargement dependent thereon.

Well, why did he not go on thus? Why did not the œdma increase, and the dyspnœa increase, and he remain in the condition in which he is now? Well, at that time there were undoubtedly excesses which impaired his general health. He was run down for some reason or other; anæmic, or his powers impaired, and under those circumstances the heart affection produced these effects. But he probably laid up, or took more care of himself, and improved his general condition, and then the heart went on again pretty well, so that he was able to continue his work, and which, as you see, was pretty hard work, involving a good deal of exposure. But still he was able to go on with that work. Then again, a year afterwards, some other excesses probably occurring, he again suffered from dyspnœa and œdema, and then palpitation of the heart troubled him, and he had cough, but after a few weeks this disappeared. Thus the history shows very strikingly how cardiac affections are tolerated, provided the condition of the system be good in other respects. And we may thus often, by improving the condition of the patient, render the heart affection but little troublesome for a considerable length of time.

This patient improved, then went to work again, was perhaps imprudent in certain ways, at all events pursued his business, which involved hard work and a good deal of exposure. Then six and a half months ago, he again had œdema of the face, of the feet and legs, and now he entered the hospital.

Well, his present condition, as you see, is not a very good one. He is pallid, he is feeble; the œdema has diminished since he has been in the hospital, but it is not all gone; any exercise doubtless would cause dyspnœa.

Now, what are the indications for treatment? We can not remove his cardiac affection. The mitral insufficiency has to remain. We cannot even diminish it. We cannot reduce the size of the heart. It would not be an object for us to do it if we could. So far as the heart is concerned, what we should aim to do would be to increase the vigor, the contraction, and we would do that permanently, not by any remedies, but by improving his general condition. Give the patient, if possible, good blood; it is poor, as you see by his aspect. The indications for treatment, then, relate to the system at large, and to the heart indirectly. There is little indication here for digitalis. The heart acts regularly. It is not remarkably feeble. And by the way, this remedy, digitalis, which is so very valuable in some cases, is often abused I may say. That is, the idea of some seems to be that whenever a patient has trouble of the heart digitalis is the remedy. Now it is a most valuable remedy in certain circumstances, but by no means a good remedy in other circumstances. Where the heart is irregular in its action as well as enfeebled, it acts sometimes in a remarkably good manner; gives the heart greater regularity and greater strength, but that is all temporary. What is desirable in this case is, that the patient's general condition be improved if possible. Tonics to improve the appetite, if that be deficient; tonic remedies to promote digestion, if that be weak or disturbed; a nutritious diet. These constitute the measures of treatment in such a case as this. Shall we try to get rid of the little œdema which now exists? This œdema does not occasion any inconvenience. He has not enough water in the chest, in the abdomen, or in the skin to be a source of any great inconvenience. Therefore we should not give the patient hydragogues and diuretics which interfere with nutrition, and would therefore do more harm than good. Our object is to improve the condition of the blood in this case, and if that can be accomplished, if the patient can be given a good appetite, good digestion, and a better quality of blood, it is very likely that with even the amount of disease which he has, he will suffer little inconvenience from his cardiac affection.

The next patient whom I should have brought in had I had time, is a case that presents a very striking and a very instructive contrast to this case. In the other patient you would see that while there is as much enlargement probably as in this man's heart; while there is the same lesion, the same murmur, yet that patient is knocking about the hospital, and very likely will want to go out of the hospital very soon. He presents the picture of comparative health. Why? I assume that their cardiac affection is the same; it would be perfectly consistent with clinical experience that it should be so; but in one case we have anæmia, deficient appetite, weak digestion, general feebleness; in the other case we have a good condition of the blood, good appetite, good digestion, and the patient is able to tolerate his affection without much inconvenience; whereas, in the case which has been brought before you, owing to his general condition, the heart affection gives the patient trouble and inconvenience.

